# Impact of Frailty on Healthcare Outcomes after Cardioembolic Ischaemic Stroke Due to Atrial Fibrillation

**DOI:** 10.3390/ijerph21030270

**Published:** 2024-02-27

**Authors:** Rónán O’Caoimh, Laura Morrison, Marion Hanley, Caoimhe McManus, Kate Donlon, Patricia Galvin

**Affiliations:** 1 Department of Geriatric and Stroke Medicine, Mercy University Hospital, Grenville Place, T12 WE28 Cork City, Ireland; 2 Health Research Board Clinical Research Facility, University College Cork, Mercy University Hospital, T12 WE28 Cork City, Ireland; 3 Department of Stroke Medicine, University Hospital Galway, Newcastle Rd, H91 YR71 Galway City, Irelandmarion.hanley@nohc.ie (M.H.);

**Keywords:** stroke, atrial fibrillation, frailty, hospital, mortality, length of stay, disability, rehabilitation

## Abstract

Stroke due to atrial fibrillation (AF) is more common in older adults. Frailty is associated with AF. As little is known about the impact of frailty on cardioembolic stroke, we examined its association with important healthcare outcomes including mortality and functional outcome in stroke with AF. Data were collected from patients presenting consecutively to a regional university hospital to assess pre-admission frailty using the Clinical Frailty Scale (CFS) and function with the Modified Rankin Scale (mRS). Stroke severity was assessed on the National Institute of Health Stroke Scale (NIHSS). In total, 113 patients presenting between August 2014 and July 2016 were identified with cardioembolic stroke, median age 80 years; 60% were male. Their median NIHSS score was 6. The median pre-admission CFS score was 3; 26.5% scored ≥5/9, indicating frailty. The median pre-admission mRS scores increased significantly from 1 to 3 at discharge (*p* < 0.001). Frailty was associated with worse mRS scores at discharge, odds ratio 1.5, (*p* = 0.03). While no patients with frailty were suitable to avail of early supported discharge, 10% of those without frailty were (*p* = 0.02). There was no significant difference in 30-day mortality. Frailty is prevalent among patients with cardioembolic stroke due to AF and was associated with poorer functional outcomes. Although the numbers were small, these data suggest that brief frailty assessments are useful to risk-stratify patients with acute cardioembolic stroke. Frailty status on admission with stroke due to AF can help identify those more likely to have poorer outcomes, to benefit from intervention, to require prolonged rehabilitation, and to avail of ESD.

## 1. Introduction

Frailty is an age-associated syndrome characterised by decreased resilience to stressors that is attributed to cumulative, multi-system decline resulting in an increased vulnerability to adverse healthcare outcomes [1]. It has a high prevalence globally with estimates varying from approximately 12% using physical definitions of frailty to 24% using the deficit accumulation model among community dwellers aged ≥50 years [2]. Frailty is a predictor of all-cause mortality [3] and is associated with an increased risk of death in patients discharged from acute care settings [4]. It is also associated with increased odds of hospitalisation and functional decline [5]. Stroke risk increases with age [6], with the prevalence in older people estimated to be 7.4% globally [6]. Cardioembolic ischaemic stroke due to atrial fibrillation (AF) is a common mechanism, particularly among older adults [7,8]. AF predicts greater stroke severity [9], and older patients with cardioembolic stroke have increased disability and higher mortality rates [10]. Further, age and greater stroke severity predict the likelihood of worse outcomes including greater mortality following stroke [10]. At population level, the ageing of society is expected to increase the risk of AF and hence, cardioembolic stroke in turn increasing the burden of care and economic costs [8]. Given the growing awareness of the role frailty plays in the increasing risk of adverse outcomes in older people, it is important to better understand its impacts on stroke care and survival, especially in the most severe strokes, i.e., cardioembolic stroke due to AF.

Frailty is associated with an increased incidence of stroke [11] and results in higher rates of mortality [11,12], LOS in hospital [11], and institutionalisation among stroke patients [13]. As the severity of frailty increases, mortality after stroke increases independent of other risk factors [12]. Frailty is also common in people with AF [11,14,15]. Prevalence rates are high but vary widely by population characteristics with estimates ranging from 48.2 to 75.4% [14]. Frailty increases both the risk of stroke and AF [15]. Although the exact mechanism by which frailty contributes to the pathophysiology of cerebrovascular and cardiovascular disease is unknown, several concepts and theories are proposed [16]. Primarily, the reduction in physical fitness characteristics of frailty may impact upon the ability to address and modify vascular risk factors such that patients may have reduced exercise tolerance and conditions, such as obesity, and hypertension diabetes and hypercholesterolemia may be less well managed in frailty [16]. Brain frailty, reflecting small-vessel white matter ischaemic disease has also been described and been associated with frailty and progression in physical frailty, potentially suggesting a bi-directional relationship [17]. Frailty can also impact negatively on decisions to treat risk factors for AF and to anticoagulate older patients with AF [18,19,20]. This likely reflects a true increased risk with treatment and a degree of therapeutic nihilism, which in turn compounds poor control of risk factors for stroke and AF. In a nationwide survey of primary care records in England, frailty was associated with an increased risk of gastrointestinal bleeding in those eligible for anticoagulation for AF [15]. In a sample of older patients in long-term care, frailty scores were associated with low rates of anticoagulation despite a very high risk of stroke among those with AF [18]. In a systematic review and meta-analysis, being labelled as frail was associated with lower odds of receiving appropriate anticoagulation. Specific markers of frailty such as walking speed and grip strength pre-dispose to reduced survival and a poorer recovery after stroke among older adults and could be used to identify those likely to have more complex acute admissions [21]. Hence, frailty and surrogate markers for this may help identify individuals with stroke and AF that will benefit from bespoke management strategies and tailored interventions, incorporating both acute medical care and post-acute rehabilitation to maximise survival and recovery, respectively [21].

Despite these points, little is known about the impact of frailty on healthcare outcomes after cardioembolic stroke due to AF or which factors may influence this association. This is important as oral anticoagulation, particularly with direct oral anticoagulants, markedly reduces the risk of stroke including secondary events with AF [22]. Understanding if frailty could improve clinical decision making or identify patients more likely to experience adverse healthcare outcomes in this setting is therefore important. The objective of this study was to examine the impact of frailty on clinical recovery, LOS, and 30-day inpatient mortality post-stroke with AF.

## 2. Materials and Methods

### 2.1. Data Collection

We conducted a retrospective cohort study using data from a single, large university hospital in the west of Ireland, which operates a regional stroke service. Patients were identified from an existing database of stroke patients presenting consecutively over an approximately two-year interval from August 2014 to July 2016. Stroke subtype was classified as per TOAST (Trial of Org 10,172 in Acute Stroke Treatment) criteria to identify stroke subtypes [23]. All cardioembolic ischaemic strokes due to AF were extracted from the dataset and entered into an electronic database for analysis. Large artery atherosclerotic sources of thrombo-embolism were excluded by either carotid doppler imaging or CT angiography. AF was confirmed from medical records, discussion with primary care providers, or, if undiagnosed prior to the stroke, by electrocardiogram or a Holter cardiac monitor recording. Those without AF were excluded. No other exclusion criteria were applied. A retrospective chart review was performed for each patient identified as having an ischaemic stroke due to AF, and pre-specified outcome measures were obtained. This is a secondary analysis of a larger studying examining stroke and bleeding risks. Ethics approval was granted in advance from the Clinical Research Ethics Committee of Galway University Hospitals, reference number C.A 1609.

### 2.2. Measures

Pre-admission frailty status was assessed using the nine-point Clinical Frailty Scale (CFS) taking a cut-off of ≥5 for established frailty [24]. The CFS has previously been studied in patients after stroke [11]. All patients were stratified based on their pre-morbid functional and cognitive status based on a retrospective chart review and consensus of two trained raters, a validated reliable approach to ascertaining frailty [25]. Baseline functional status and post-stroke functional level (i.e., immediately at discharge or last available functional assessment prior to discharge) were measured with the Modified Rankin Scale (mRS) score, a widely used measure of global disability ranging from “no symptoms” (score of zero) to “death” (score of six) [26]. Stroke severity was assessed using the National Institute of Health Stroke Scale (NIHSS) score, an 11-item, reliable stroke deficit rating scale scored from 0 to 42, ranging from no deficit to the most severe stroke, with scores < 4 suggesting better outcomes [27]. The NIHSS score is a predictor of mortality and risk of recurrent stroke [10]. Frailty prior to stroke is associated with stroke severity based on the NIHSS [28]. The CHA_2_DS_2_VASc [29], a clinical prediction tool measuring annual stroke risk with (non-valvular) AF and HAS-BLED, a clinical risk score assessing one-year risk of major bleeding in AF [30], were independently calculated for each patient. The CHA_2_DS_2_VASc score comprises seven variables, all related to stroke risk factors [29]. There is one point given for a history of congestive heart failure, hypertension, female sex, known diabetes, established hypertension, history of vascular disease, and age between 65 and 74 years [29]. A score of two points is given for those aged 75 and older and for those with a past history of a stroke or transient ischaemic attack [29]. Generally, a score of one for males or a score of two for females is considered as a low to moderate risk and anticoagulation can be considered. The CHA_2_DS_2_VASc score is a widely used risk prediction tool and has been used in patients with frailty to support decisions to anticoagulate adults with AF [18]. HAS-BLED also includes seven variables (age ≥ 65 years, alcohol use, medication use predisposing labile INR measures, history of stroke, past history of bleeding or an existing predisposition to bleeding, uncontrolled hypertension, and presence of liver disease and underlying chronic kidney disease) with each given the same weighting (i.e., one point) [30]; a total HAS-BLED score of three or greater indicates a high risk of bleeding with anticoagulation for AF [30]. HAS-BLED has also been used in patients with frailty to support clinical decision making [18].

### 2.3. Outcomes

LOS including time in rehabilitation and 30-day mortality were calculated from national hospital administrative data (Hospital In-Patient Enquiry Scheme). Rates of uptake and suitability for early supported discharge for stroke (ESD) [31], provided they were within the hospital catchment [32], were recorded from medical notes and or discussion with the ESD service. Prolonged LOS was taken as an admission duration of >14 days, the median LOS in Irish hospitals after stroke [33].

### 2.4. Data Analysis

Data were analysed using SPSS version 24.0 (SPSS Inc., Chicago, IL, USA) and R version 4.2.2 (31 October 2022)—“Innocent and Trusting” (R Core Team, 2022). All significance tests were two-sided, and a *p*-value of <0.05 was considered statistically significant. Most data were non-normally distributed and analysed with non-parametric tests. Spearman’s rho (*r*) measured rank correlation. The Chi-squared test assessed differences between the distributions of categorical variables. The Mann–Whitney U test and the Kruskal–Wallis test were used to examine differences between non-parametric continuous variables. Frailty was categorised into two groups: frail and non-frail using a cut-off of ≥5 on the CFS to denote frailty [24], as this is widely used in the measurement of frailty in those with stroke [34]. Logistic and multiple linear regression were performed to examine if frailty status was an independent predictor of adverse outcomes.

## 3. Results

### 3.1. Baseline Characteristics

In total, 113 patients were identified with ischaemic stroke and AF. Of these, the median age was 80 years, interquartile range (IQR) ±14, range 54–101 years, and most, 60%, were male. The median pre-admission CFS score was 3 (IQR ± 3), and 26.5% (n = 30) scored ≥5, indicating that most were mildly to severely frail. Patients classified as frail were statistically significantly older, *p* = 0.008, and a greater proportion were female (67% versus 30%), *p* = 0.001. Frailty (baseline CFS scores) correlated most closely with patient age (r = 0.42, *p* < 0.001) and patient post-stroke mRS scores (r = 0.41, *p* < 0.001). The median pre-admission mRS score for all patients was 1 (±2) increasing to a median of 3 (±2) at discharge, z = −7.5, *p* < 0.001. Patients categorised as frail had significantly worse scores at baseline, and at discharge; the scores at discharge were 4 versus 3 for those classified as frail versus non-frail, *p* < 0.001. The median baseline mRS was 3 for frail patients, which increased to a median of 4 post-stroke. The median NIHSS score for all patients was 6 (±8). There was a trend towards patients with frailty having higher baseline NIHSS scores, 7.5 vs. 5, *p* = 0.09, although this did not reach statistical significance. There was no difference in NIHSS scores by baseline mRS score (*p* = 0.21). The median CHA_2_DS_2_Vasc score was 4 (±2), and patients categorised as frail had statistically significantly higher scores, *p* = 0.02. There was no significant different in median HAS-BLED scores, *p* = 0.26. The characteristics of patients included is presented in Table 1.

### 3.2. Stroke Outcomes

The median LOS in hospital for all patients was 17 (±19) days, increasing to 21 (±43) when time in off-site rehabilitation was included. Frail patients (CFS ≥ 5) had a longer acute hospital LOS compared to those not classified as frail, median 22 vs. 15 days, *p* = 0.04. In all, 33.6% were transferred for rehabilitation to an off-site stroke rehab unit of whom 12.5% were classified as frail at baseline. There was a trend towards a longer total (including rehabilitation time), 28 versus 20 days, respectively, though this did not reach statistical significance, *p* = 0.14. The overall 30-day mortality rate for the total sample was 10% (11/113). There was no significant difference in 30-day mortality between those identified as frail compared to non-frail (*p* = 0.95). In total, 10% of non-frail but none (0%) of the frail patients were suitable and agreeable to avail of ESD, *p* = 0.02.

### 3.3. Regression Analysis

After adjusting for possible confounding variables by including age, male sex, NIHSS score, HAS-BLED and CHA_2_DS_2_Vasc scores in modelling, frailty status (based on baseline CFS) was associated with worse functional outcomes after stroke with higher frailty scores independently predicting post-stroke mRS scores, odds ratio (OR) 1.5, and 95% confidence interval (CI):1.04–2.1 (*p* = 0.03). NIHSS scores were associated with lower odds of a change in mRS scores and OR 0.86 (95% CI: 0.78–0.96, *p* = 0.007), possibly reflecting that those with a higher mRS at baseline (≥3) had more severe strokes (mean NIHSS 9.0 vs. 7.3). These data are presented in Table 2.

Frailty was not found to be an independent predictor of hospital LOS (*p* = 0.43), total LOS (including formal rehabilitation time) (*p* = 0.32), or mortality (*p* = 0.80). NIHSS scores, indicating stroke severity, were an independent predictor of mortality, OR of 4.0, and 95% CI: 1.2–13.4, (*p* < 0.001). NIHSS scores were also independently associated with LOS incorporating time in rehabilitation, OR of 1.30 and 95% CI: 0.50–2.6, (*p* = 0.011). None of the variables were identified as independent predictors of increased LOS in the acute hospital (excluding time spent in rehabilitation).

## 4. Discussion

This study examined the impact of frailty on functional recovery and short-term hospital outcomes (LOS in acute care including rehabilitation time and 30-day mortality) among patients with cardioembolic stroke associated with AF. To our knowledge, it is the first study to specifically examine the impact of frailty on stroke due to AF as the primary identified cause. Patients who were frail prior to their stroke due to AF were older and female and had more stroke risk factors than those who were not characterised as frail. The results show that pre-admission frailty status was an independent predictor of functional decline based on the mRS but did not predict other outcomes when potential confounders were considered. Rather, it was stroke severity as measured by the NIHSS that significantly predicted short-term mortality and LOS when it included time spent in rehabilitation. Patients identified as frail had a median post-stroke mRS of 4 compared to a median post-stroke mRS of 3 in those without frailty, reflecting that baseline frailty is strongly associated with activities of daily living and may indicate pre-existing (baseline) functional decline. This is clinically meaningful as a change of one mRS grade is considered to be clinically significant [35]. In reality, this corresponds to a much higher degree of dependence. An mRS of 4 indicates moderately severe disability, with a patient requiring assistance for walking and not being able to attend to their own bodily needs without assistance [26]. This supports previous studies that have shown that markers of frailty such as a slow walking speed and grip strength predict poor recovery post-stroke [13,21]. There is also extensive evidence that stroke severity as measured by the NIHSS is a key predictor [36], albeit it requires careful training to be accurate in clinical practice [37]. Understanding the biological mechanisms underlying the contribution of frailty to cardioembolic stroke is important to address measures to reduce the impact of stroke due to AF [16]. That those with established frailty had significantly higher CHA_2_DS_2_Vasc scores at baseline likely reflects the overall vascular burden among those with frailty [16]. This in turns increases the risk for cerebrovascular disease in those with frailty, likely increasing stroke risk [17]. Baseline small-vessel microangiopathic disease is more prevalent In stroke and predicts a worse prognosis after stroke [17].

Patients who have cardioembolic ischaemic strokes generally have worse outcomes, due to increased stroke severity and higher mortality rates [38,39,40]. This is independent of frailty status. In this study, we did not find any difference in 30-day mortality between those with and without frailty. This may be because although frailty predicts long-term mortality after hospitalisation with stroke [12], there is as yet mixed evidence that it is associated with short-term inpatient mortality in this setting [41]. While the numbers were small and non-significant in the multi-variate analysis, the data show a trend towards an association between frailty and increased LOS in an acute hospital and subsequent time in rehabilitation in older patients with ischaemic stroke and AF. However, again, this is likely explained by stroke severity resulting in more complex and prolonged admissions [42,43]. Patients with frailty are more likely to require inpatient rehabilitation for longer periods of time.

Reflecting this, patients characterised as having frailty were less likely to avail of ESD. ESD, centred on the provision of multi-disciplinary team-based therapy at home, is best targeted to stroke survivors with mild to moderate disability; ESD improves functional gains and reduces rates of institutionalisation [42,44]. Patients who do not receive ESD often stay longer in hospital, so it is important to highlight that patients with cardioembolic stroke who are also frail are generally not deemed to be candidates for ESD. Patients who receive ESD services are more likely to be independent and living at home after a stroke than those who received conventional services [42]. That no patients in our study with frailty were deemed suitable for ESD likely reflects the increased disability post-stroke found in those with frailty. This suggests that in an era of increasing co-morbidity and ageing populations, patients with frailty may be more likely to receive all of their stroke rehabilitation in an inpatient setting, which is important for service planning in the future. Frailty assessments may improve risk prediction for patients at risk of increased LOS post-stroke. When considering the economics of stroke, rehabilitation services are the main driver of post-stroke care costs [45]. Hence, including an assessment of frailty at admission may help to plan delivery of care and allocation of resources by predicting early those most likely to need formal inpatient rehabilitation [46,47]. Patients who have a stroke and are frail have a lower self-rated quality of life both during the acute phase of the admission and during the prolonged follow-up, emphasising the need to better assess and understand frailty in stroke units [48]. This is important as frailty may contribute to worse rehabilitation outcomes for these patients including reduced mobility and a poorer mood [49]. Routine screening in acute care may provide better personalised treatment strategies including bespoke rehabilitation and advance care planning to minimise the impact of frailty in this group [48]. Indeed, as there can be tensions around the need to make rapid decisions to identify who may benefit most from rehabilitation, understanding the role frailty has in the course of these patients’ admission and subsequent recovery is paramount [50]. Here, we have shown the need to identify frailty early upon admission, even with relatively simple instruments such as the CFS and other similar brief screens, which can be performed quickly on arrival to hospital, e.g., to the emergency department [51]. Instruments such as the CFS have the potential to identify those at a higher risk both on admission and discharge from rehabilitation units with increases in these scores being associated with three-month and one-year mortality [52]. In our study, while CFS scores increased from pre-admission to discharge, we did not see any association with mortality; albeit we only followed outcomes to 30 days, i.e., inpatient mortality.

This study is also one of the first to examine the interaction between the NIHSS and frailty. Frailty prior to stroke is statistically significantly associated with NIHSS scores with an increase in frailty resulting in more severe stroke as measured by the NIHSS [26]. In this study, while NIHSS scores (reflecting stroke severity) were higher in those with frailty, this was not statistically significant. In addition, those with higher baseline disability as measured by the mRS had more severe strokes per the NIHSS, which may in part reflect frailty. Frailty remained independently associated with functional status after cardioembolic stroke, as reflected by the mRS. This has been found in another study, which included generic strokes without subtypes. Authors found that in a regression analysis when potential confounders were adjusted for, frailty (physical frailty based on the FRAIL scale), was significantly associated with post-stroke disability based on the mRS [53]. The relationship between frailty and the NIHSS is important as deciding to provide thrombolysis or proceed with mechanical thrombectomy (MT) in stroke is supported by NIHSS scores and how frailty impacts on its scoring may influence the clinical decision. Indeed, each one-point increase in CFS is associated with a reduction of one point in the NIHSS after ischaemic stroke [12]. That frail and non-frail patients have similar rates of thrombolysis and MT, but that those that are frail have poorer outcomes independent of the NIHSS scores [54,55], suggests that it may be less useful in this setting and that measures of frailty may be more relevant alone or in combination with traditional indicators of risk and benefit. However, at present little is known about if and how the scoring of the NIHSS should be adapted for patients with frailty at baseline. Similarly, it is not known if frailty should change the management of stroke based on NIHSS scoring.

Limitations of this study include its retrospective nature, the lack of follow-up data beyond the present admission, limiting inferences, and the small sample size, likely under-powering the study. This was a retrospective cohort study examining consecutive strokes attending the hospital over a two-year period, and a sample size was not calculated a priori. Further, as the data collection was limited to 2014–2016, the results may be less generalisable to recent clinical practice. This said, frailty is only recently being widely recognized as an accurate risk stratification approach to tailor appropriate management to patients with stroke, irrespective of age [56], and this is first study to examine its impact on stroke due to AF; the passage of time is not likely to alter the importance or relevance of these findings. Although small, it has the advantage of having a complete case ascertainment, given that stroke registry data are complete, as mandated nationally by Ireland’s National Clinical Programme for Stroke, which oversees the collection of stroke data in Ireland since the National Stroke Register was developed here in 2013 [57]. Another limitation is that only a single measure of frailty was used, which was based on information available in the chart rather than on a detailed comprehensive geriatric assessment (CGA), meaning that patients may have been misclassified or would have been classified differently based on other definitions of frailty, e.g., using the frailty phenotype. The chart-abstracted version of the CSF was used, which has been utilized in previous studies and is shown to be reliable [25] and accurate in other settings [47]. We did not have data on the proportion of patients who were taking and compliant with therapeutic anticoagulation, limiting our analysis and the ability to examine another potential confounder. An additional limitation is the use of 15 days or more (>14) to represent prolonged LOS. This was selected as it is specific to post-stroke LOS in Ireland [33]. The median LOS in Irish hospitals is lower than this, and other studies usually apply non-disease specific LOS values to denote this outcome, potentially reducing comparability with other studies and generalizability in clinical practice. Finally, there is a risk of misclassification bias as it is possible that some strokes attributed to AF may have been due to other causes, although the diagnosis was based on a detailed clinical work-up, which included cardiac monitoring and excluded other aetiologies, and all strokes were labelled as due to AF in the national register.

## 5. Conclusions

This study is the first to examine the impact of frailty on individuals with stroke due to AF. It found that patients who were characterised as frail were statistically significantly more likely to be older, female, and have more risk factors for stroke as measured by the CHA_2_DS_2_VASc score. The results of this study support the hypothesis that frailty is a proxy measure for patients likely to experience poorer functional recovery after ischaemic cardioembolic stroke due to AF as measured by changes in the mRS. Frailty may compound post-stroke cognitive and functional impairment and stroke severity, limiting the potential to return to baseline status as reflected by the mRS. Although numbers were small, likely under-powering the study, these initial data suggest a trend towards an association between frailty and increased LOS. The study also highlighted that these patients are less likely to avail of ESD. Thus, a frailty assessment on admission, even with brief instruments such as the CFS, represents an important approach to aid prognostication after stroke due to AF, especially for those likely to need formal inpatient rehabilitation. Further suitably-powered studies are now needed to better characterise the relationship between frailty and stroke due to AF, to examine for long-term outcomes and to explore if optimal management approaches targeting frailty can improve outcomes in this population. Further study is also now required to better understand risk factors that lead to increased vulnerability to adverse healthcare outcomes in patients with frailty and stroke due to AF. Understanding these factors will help to optimise recovery and long-term outcomes in this population. Further research is also needed to understand how frailty impacts on the use of stroke scales such as the NIHSS to ensure that they are appropriate outcome measures for these patients. Finally, future research should also focus on understanding the pathophysiological mechanisms of how frailty is associated with the development of stroke and AF as well as how it impacts on outcomes.

## Figures and Tables

**Table 1:** Characteristics and summary of healthcare outcomes comparing frail and non-frail patients with stroke secondary to atrial fibrillation (AF) based on the Clinical Frailty Scale (CFS) scores.

Variable	Total (n = 113)	Frail (CFS ≥ 5) (n = 30)	Non-Frail (CFS ≤ 4) (n = 83)	*p* = X
Age	80 (+/−14)	82.5 (+/−10.7)	78 (+/−12.5)	*p* = 0.008 *
Sex (% Male)	68/113 (60%)	10/30 (33%)	58/83 (70%)	*p* = 0.001 *
NIHSS score	6 (+/−8)	7.5 (+/−8.5)	5 (+/−7.5)	*p* = 0.09
Pre-Stroke mRS	1 (+/−2)	3 (+/−0)	1 (+/−1)	*p* < 0.001 *
Post-Stroke mRS	3 (+/−2)	4 (+/−1)	3 (+/−1)	*p* < 0.001 *
CHA_2_DS_2_Vasc	4 (+/−2)	4.5 (+/−2)	4 (+/−2)	*p* = 0.02 *
HAS-BLED	2 (+/−1)	2 (+/−2)	2 (+/−1)	*p* = 0.26
Length of stay (acute days)	17 (+/−19)	22 (+/−30)	15 (+/−16)	*p* = 0.04 *
Length of stay (total days **)	21 (+/−43)	28 (+/−61)	20 (+/−40)	*p* = 0.14
Suitable and availed of ESD	8/113 (7.1%)	0/30 (0%)	8/83 (10%)	*p* = 0.02 *
30-day mortality rate	11/113 (10%)	3/30 (10%)	8/83 (10%)	*p* = 0.95

CFS = Clinical Frailty Scale; ESD = Early Supported Discharge; mRS = Modified Rankin Scale; NIHSS = National Institute of Health Stroke Scale; * Statistically significant; ** Including time in rehabilitation hospital.

**Table 2:** Binary logistic regression model showing the association between variables and worsening (increase between pre-stroke and post-stroke indicating worsening disability) Modified Rankin Scale scores.

Variable	Odds Ratio	95% Confidence Interval	*p* = x
Age	0.95	0.89–1.0	0.07
CHA_2_DS_2_Vasc	1.07	0.76–1.52	0.68
CFS score	1.5	1.04–2.07	0.03
HAS-BLED	1.08	0.60–1.91	0.80
NIHSS	0.86	0.78–0.96	0.007
Sex (male)	0.63	0.22–1.78	0.39

CFS = Clinical Frailty Scale; NIHSS = National Institute of Health Stroke Scale.

## Data Availability

Data are available on request.
